# Potent and Selective Activity against Human Immunodeficiency Virus 1 (HIV-1) of *Thymelaea hirsuta* Extracts

**DOI:** 10.3390/v12060664

**Published:** 2020-06-19

**Authors:** Giuseppina Sanna, Silvia Madeddu, Giuseppe Murgia, Gabriele Serreli, Michela Begala, Pierluigi Caboni, Alessandra Incani, Gianluigi Franci, Marilena Galdiero, Gabriele Giliberti

**Affiliations:** 1 Department of Biomedical Sciences, University of Cagliari, Cittadella Universitaria, Monserrato, 09042 Cagliari, Italy; silvia.madeddu@unica.it (S.M.); gabriele.serreli@unica.it (G.S.); elisa.incani@gmail.com (A.I.); gabrielegiliberti@hotmail.com (G.G.); 2 Department of Inorganic and Analytical Chemistry, University of Cagliari, Cittadella Universitaria, Monserrato, 09042 Cagliari, Italy; murgiape@tiscali.it; 3 Department of Life and Environmental Sciences-Unit of Drug Sciences, University of Cagliari, Cittadella Universitaria, Monserrato, 09042 Cagliari, Italy; michelabegala@unica.it (M.B.); caboni@unica.it (P.C.); 4 Department of Medicine, Surgery and Dentistry “Scuola Medica Salernitana”, University of Salerno, 84081 Baronissi, Italy; gfranci@unisa.it; 5 Department of Experimental Medicine, University of Study of Campania “Luigi Vanvitelli”, Via Costantinopoli 16, 80138 Napoli, Italy; marilena.galdiero@unicampania.it

**Keywords:** thymelaea hirsuta extracts, anti-HIV-1 activity, HIV resistant mutants, lactobacilli, TEER

## Abstract

Historically, natural products have been the most successful source of inspiration for the development of new drugs. Members of the Thymelaeaceae family have been of interest owing to their excellent medicinal value. Given the successful history of natural product-based drug discovery, extracts from the aerial parts of *Thymelaea hirsuta* were evaluated for their potential anti-human immunodeficiency virus type 1 (HIV-1) activity. Ethyl acetate extracts from leaves (71B) and branches (72B) of *Thymelaea hirsuta* showed potent and selective activity against HIV-1 wt (EC_50_ = 0.8 µg/mL) at non-cytotoxic concentrations (CC_50_ > 100 µg/mL). They proved to be active against HIV-1 variants carrying clinically relevant NNRTI and NRTI mutations at low concentration (0.3–4 µg/mL range) and against the M-tropic strain HIV-1 BaL. The 72B extract, chosen as a lead, was not able to inhibit the RT and protease enzymatic functions. Furthermore, it was not virucidal, since exposure of HIV to high concentration did not affect virus infectivity. The pre-clinical safety profile of this extract showed no adverse effect on the growth of Lactobacilli, and non-toxic concentration of the extract did not influence the Caco-2 epithelial cells monolayer integrity. Additionally, extract 72B prevented syncytia formation at low concentration (0.4 µg/mL). The potent inhibitory effect on the syncytia formation in co-cultures showed that 72B inhibits an early event in the replication cycle of HIV. All of these findings prompt us to carry on new studies on *Thymelaea hirsuta* extracts.

## 1. Introduction

In olden times, the most important source of inspiration for the development of new drugs hasbeen natural compounds of plant origin [1]. Indeed, the high chemical diversity and biochemical specificity in their structures make them ideal lead candidates for drug discovery [2]. Moreover, investigations showing the antiviral potential of plant extracts against viral strains resistant to conventional antiviral agents [3,4] have challenged the recent drug discovery techniques, deeming a very careful look toward exploring natural antiviral components of medicinal plants. Since ancient times, phytotherapy has a considerable role in sustaining and improving the health and quality of human life. The World Health Organization estimates that 80% of people living in developing countries depend on traditional medicinal practices for their primary health care. Medicinal plants are even more used in countries where the health system does not guarantee high levels of efficiency and in economically more fragile countries.

The literature search reveals that members of the Thymelaeaceae family have been of interest owing to their excellent medicinal value [5]. The beneficial effects are strongly linked to their content in flavonoids, coumarins, and diterpenoids, which are some of the compounds most frequently isolated from these types of plants [6]. Given the successful history of natural product-based drug discovery, we then focused our attention on *Thymelaea hirsuta*, a perennial, evergreen, shrub, growing up to 2 m tall that commonly grows in Sardinia, in the Mediterranean coastal plains, the Sinai Peninsula and other Sahara-Arabian deserts. Thanks to its antimelanogenic activity [7], and its antioxidant [8], hypoglycaemic and antidiabetic properties [9], *T. hirsuta* has been often used in traditional medicine. Moreover, extracts from *T. hirsuta* have recently shown anti-inflammatory and antiarthritic activity [10], as well as antidiabetic and antihypertensive activity, due to their rich polyphenol content [11], and antitumor and antioxidant activity [12], also due to their polyphenol content [13]. In Sardinia, the infusion made with the leaves has been, in traditional medicine, used for the treatment of edema and the mash obtained from the leaves of *Thymelaea hirsuta*, *Verbena officinalis* and olive oil has been used to treat herpes [14].

The main components of plants of the Thymelaeaceae family are daphnane diterpenes and coumarins, as well as lignans and phenolics [15]. Daphnane diterpenes are the major biologically active constituents of Thymelaeaceae. A large number of them were extracted and isolated in recent years from different species of Daphne, such as *D. gnidium* [16], *D. genkwa* [17], *D. acutiloba* [18], as well as from *Thymelaea hirsuta* [19]. Daphnane-type diterpenes exhibit a wide range of biological activities, such as antioxidant [20,21], antiflea insecticidal [22,23], antileukemic [24], neurotrophic [25], antihyperglycemic [26] activity. Moreover, daphnane-type diterpenes showed proliferating inhibitory activity against human cancer lines [27]. Additionally, significant anti-HIV-1 activities were observed in daphnane-type diterpene [28,29,30,31], indicating that daphnane-type diterpenes could be considered promising molecules for anti-HIV drug development. However, while extracts isolated from other plants belonging to the Thymelaeaceae family, such as *Daphne g*., are also known for antiviral activities against HIV-1, enterovirus 71 [32], Coxsackievirus B5 [33], SARS-CoV [34], influenza virus [35], and HBV [36], no antiviral activity related to extracts from *Thymelaea hirsuta* until now has been described. All these findings prompted us to investigate the potential antiviral properties of these extracts.

In the present study, natural extracts derived from leaves and branches of *Thymelaea hirsuta* were assessed in cell-based assays and we report for the first time their selective and potent anti-HIV-1 activity.

## 2. Materials and Methods

### 2.1. Plant Materials

*Thymelaea hirsuta* was collected from the “Forest Station and Environmental Surveillance” of Senorbì in the countryside of Samatzai (Sardinia, Italy) between March and October of 2008. Extracts were obtained from branches and leaves. A voucher specimen of the plant is kept at the Department of Biomedical Sciences, University of Cagliari (Italy).

### 2.2. Flash Extraction with Funnel Büchner

The dried and powdered plant materials (approximately 80 g) were extracted with acetone (500 mL). The pooled extracts were evaporated under vacuum. The extracts were added to silica gel (SiO_2_, 230–400 mesh) to get a powder. Inside the Büchner funnel, associated with a vacuum flask (connected to a vacuum pump), were added in sequence: a layer of SiO_2_, filter paper, a layer of celite, filter paper, and finally the silica dust containing the extracted organics. The extraction is done in four steps, using four increasing polarity solvents, in the following order: *N*-hexane, ethyl acetate (EtOAc), and acetone (200 mL of each solvent), giving the extracts A, B, and C respectively. After complete removal of the solvent, the extracts previously weighed, were evaluated for biological activity.

### 2.3. Cells and Viruses

The cell line supporting the multiplication of HIV-1 was CD4+ human T-cells containing an integrated HTLV-1 genome (MT-4); the human T cell line was immortalized by human T cell leukemia virus (C8166). PM1 (promyelocytic cells) expressing CD4, CCR5, and CXCR4 susceptible to infection by both R5 (macrophage-tropic) and X4 (T cell-tropic) strains of HIV-1 were obtained from NIH AIDS Research &Reference Reagent Program, Germantown, MD, USA as H9/IIIB cells, an H9 cell clone which is persistently infected with HIV-1 (Popovic et al., 1984).

Madin Darby Bovine Kidney (MDBK) (ATCC CCL 22 (NBL-1) *Bos Taurus*); baby hamster kidney (BHK-21) (ATCC CCL 10 (C-13) *Mesocricetus auratus*); monkey kidney (Vero-76) (ATCC CRL 1587 *Cercopithecus Aethiops*); were purchased from American Type Culture Collection (ATCC, Manassas, VA, USA).Cell cultures were checked periodically for the absence of mycoplasma contamination with MycoTect Kit (Gibco, Carlsbad, CA, USA).

Viruses representative of positive-sense, single-stranded RNAs (ssRNA+) were: (i) Retroviridae: Human Immunodeficiency Virus type-1 (HIV-1) IIIB laboratory strain was obtained from the supernatant of the persistently infected H9/IIIB cells (NIH 1983). HIV-1 BaL, an R5 strain, was obtained from NIH AIDS Research &Reference Reagent Program, USA.The Y181C mutant (NIH N119) derives from an AZT-sensitive clinical isolate passaged initially in CEM and then in MT-4 cells, in the presence of Nevirapine (up to 10 mM). The K103N + Y181C mutant (NIH A17) derives from an IIIB strain passaged in H9 cells in the presence of BI-RG 587 (up to 1 mM). The K103R+ V179D+ P225H mutant (EFV^R^) derives from an IIIB strain passaged in MT-4 cells in the presence of Efavirenz (up to 2 mM). Mutants carrying NRTI mutations, such as the AZT^R^ strain (67N, 70R, 215F, 219Q) and the MDR strain (74V, 41L, 106A, 215Y) were also tested; (ii) Flaviviridae: bovine viral diarrhea virus (BVDV) (strain NADL (ATCC VR-534)); (iii) Picornaviridae: human enterovirus C (poliovirus type-1 (Sb-1), Sabin strain Chat (ATCC VR-1562)). Viruses representative of negative-sense, single-stranded RNAs (ssRNA−) were: (iv) Pneumoviridae: human respiratory syncytial virus (RSV) strain A2 (ATCC VR-1540); v) Rhabdoviridae: vesicular stomatitis virus (VSV) (lab strain Indiana (ATCC VR 1540)). The virus representative of double-stranded RNAs (dsRNA) was: (vi) Reoviridae reovirus type-1 (Reo-1) (simian virus 12, strain 3651 (ATCC VR-214)). DNA virus representatives were: (vii) Herpesviridae: human herpes type 1 (HSV-1) (strain KOS (ATCC VR-1493)).

Viruses were maintained in our laboratory and propagated in appropriate cell lines. The viruses were stored in small aliquots at −80 °C until use.

### 2.4. Cytotoxicity Assays

Exponentially growing MT-4 cells were seeded at an initial density of 4 × 10^5^ cells/mL in 96-well plates in RPMI-1640 medium, supplemented with 10% fetal bovine serum (FBS), 100 units/mL penicillin G and 100 µg/mL streptomycin. MDBK and BHK cells were seeded in 96-well plates at an initial density of 6 × 10^5^ and 1 × 10^6^ cells/mL, respectively, in minimum essential medium with Earle’s salts (MEM-E), l-glutamine, 1 mM sodium pyruvate and 25 mg/L kanamycin, supplemented with 10% horse serum (MDBK) or 10% fetal bovine serum (FBS) (BHK). Vero-76 cells were seeded in 96-well plates at an initial density of 5 × 10^5^ cells/mL, in Dulbecco’s modified Eagle medium (D-MEM) Gibco; Thermo Fisher Scientific, Inc., Waltham, MA, USA. with l-glutamine and 25 mg/L kanamycin, supplemented with 10% FBS. Cell cultures were then incubated at 37 °C in a humidified, 5% CO_2_, atmosphere, in the absence or presence of serial dilutions of test compounds/samples. The test medium used for the cytotoxic assay as well as for the antiviral assay contained 1% of the appropriate serum. Cell viability was determined after 72, 96 or 120 h at 37 °C by the 3-(4,5-dimethylthiazol-2-yl)-2,5-diphenyl-tetrazolium bromide (MTT) method [37].

### 2.5. Antiviral Assays

Sample’s activity against HIV-1 IIIB laboratory strain and resistant mutants was based on inhibition of virus-induced cytopathogenicity in exponentially growing MT-4 cells acutely infected with a multiplicity of infection (m.o.i.) of 0.01. Briefly, 50 µL of RPMI containing 1 × 10^4^ MT-4 cells was added to each well of flat-bottom microtitre trays, containing 50 µL of RPMI with or without serial dilutions of test samples. Then, 20 µL of an HIV-1 suspension containing 100 CCID_50_ were added. After a 4-day incubation at 37 °C, cell viability was determined by the MTT method.

Compound’s activity against Reo-1 was based on inhibition of virus-induced cytopathogenicity in BHK-21 cells acutely infected with an m.o.i. of 0.01. The activity of the compound against BVDV was based on inhibition of virus-induced cytopathogenicity in MDBK cells acutely infected with an m.o.i. of 0.01. After a 3 or 4-day incubation at 37 °C, cell viability was determined by the MTT method, as described previously. Compound’s activity against Sb-1, VSV, RSV A2, and HSV-1 was determined by plaque reduction assays in infected cell monolayers, as described previously [38]. Briefly, the monolayer of Vero-76 cells was grown overnight on a 24-well plate. The cells were then incubated with 200 µL of proper virus dilutions to give 50–100 PFU/well, immediately followed by the addition of various concentrations of the samples. The medium was also added to non-treated wells as non-infected controls. After 2 h, the inoculum was removed, and infected cells were overlaid with 1.2% methylcellulose medium containing various concentrations of test samples and incubated for 2 or 3 days at 37 °C. Then, the overlay medium was removed, and the cell monolayer was fixed with 4% paraformaldehyde solution, permeabilized, and immunostained for plaque detection. Plaques in the control (no inhibitor) and experimental wells were counted. All the experiments were conducted in triplicate and average values were plotted.

### 2.6. Anti-HIV-1 BaL Activity of 72B Extract by p24 Determination Assay

PM-1 cells were used to evaluate the anti-HIV-1 BaL activity of 72B extract.Quantitation of the p24 Gag protein present in supernatants of HIV-1 BaL infected PM-1 cells was assessed using the Alliance HIV-1 P24 ANTIGEN ELISA Kit (Perkin Elmer, Waltham, Massachusetts, USA), according to the manufacturer’s protocol, on culture supernatant using the antigen-capture enzyme-linked immunosorbent assay test (ELISA) (Perkin Elmer).

### 2.7. Virucidal Activity Assays

Cell-free, high titer HIV-1 stock solutions were exposed to test compounds (30 µg/mL for both 72B and amphotericin B) for 2 h at either 0 or 37 °C. The mixture without a test sample was used as the control. Samples were thenserially diluted in RPMI to reach a concentration of compound far below the EC_50_ and used to infect MT-4 cells. At the end of the incubation period, the residual virus infectivity was quantified by the Reed and Muench endpoint titration method.

### 2.8. H9/IIIB-C8166 Cocultures Assay

Chronically infected H9 cells were washed and cocultured with uninfected C8166 cells in the absence or the presence of test inhibitors. Following 36 h incubation at 37 °C, cocultures were monitored at different time points by optical microscopy, syncytia were counted and those found in drug-treated cocultures were reported as a percentage of those counted in untreated cocultures.

### 2.9. Transepithelial Electrical Resistance (TEER)Assay

The effect of the plant extracts on epithelial cell monolayer integrity was assessed by measuring the TEER value (transepithelial electrical resistance). Caco-2 cells (ECACC Salisbury, Wiltshire UK) were cultured in Dulbecco’s modified Eagle’s medium (DMEM), supplemented with 10% heat-inactivated bovine serum, 2 mM l-glutamine, 1% non-essential amino acids, 100 U/mL penicillin, and 100 mg/mL streptomycin, in monolayers at 37 °C in a humidified atmosphere of 5% CO_2_ [39], replacing the medium twice a week. Cell culture materials were purchased from Invitrogen (Milan, Italy).Caco-2 cells (0.5 × 10^5^ cells/well),at passage 21–40, were grown in 12 mm i.d. Transwell inserts (polycarbonate membrane, 0.4 µm pore size) (Corning Costar Corp., New York, NY, USA) and culture medium was dispensed in the basolateral compartment of each well. Resistance was measured using Millicell–ERS ohmmeter (Millicell-ERS system, Millipore, Bedford, MA, USA) as reported by Serreli et al. (2017). After the formation of a monolayer, only cells in inserts with TEER values >300 Ω/cm^2^ were considered for the experiment. Then, 72B extract (final concentration 30 µg/mL) and, as a prooxidant agent, an oxysterol mixture prepared as described by Incani et al. [40] (final concentration 60 µM) were added in the culture medium and TEER values were measured at intervals of 0.5, 1, 3, 18, 24 and 48 h and reported as percentage of the corresponding TEER value at time zero (T = 0).

### 2.10. Effect of Plant Extracts on the Viability of Lactobacilli

Selected lactobacilli strains such as *Lactobacillus casei* (ATCC 334), *Lactobacillus paracasei*(ATCC 25303), *Lactobacillus plantarum* (ATCC 14917) and *Lactobacillus rhamnosus* (ATCC 7469) were purchased from American Type Culture Collection (ATCC, Manassas, VA, USA).

The tested strains were cultured in anaerobic conditions at 37 °C for 48 h on de Man-Rogosa-Sharpe MRS agar (Oxoid) broth. All bacterial strains were stored at −80 °C in de Man Rogosa and Sharpe (MRS) medium (Oxoid, Milan, Italy) supplemented with 25% glycerol (*v*/*v*). The cultures were propagated three times with about 3% (*v*/*v*) of inoculum in MRS and incubated in anaerobiosis (AnaeroGen, Oxoid, Basingstoke, UK) overnight at 30 °C for *L. plantarum* and 37 °C for *L. rhamnosus*, *L. casei* and *L. paracasei*.

Briefly, bacterial density was adjusted to an OD of 0.06 at a wavelength of 670 nm, i.e., approximately 10^8^ CFU/mL. Extracts were administered at concentrations ranging from 2000 to 250 µg/mL into 96-well plates along with 50 µL of bacterial suspension. The final volume was made up to 100 µL using MRS broth. Negative control included cells treated with solvent/medium and Saquinavir, a known HIV-1 protease inhibitor, was used as reference control. After 24 h incubation at 37 °C, the cytotoxicity of plant extracts on lactobacilli was assessed by the MTT method.

### 2.11. Reverse Transcriptase Assay

The colorimetric reverse transcriptase assay (Roche, Cat. 11468120910) was used for the quantitative determination of retroviral reverse transcriptase activity, using efavirenz as a reference compound.

### 2.12. Protease Assay

The SensoLyte™ 520 HIV-1 protease assay kit (AnaSpec Co., Beijing, China) was used to measure HIV protease enzyme activity.

### 2.13. Statistical Analysis

Cell-based experiments were independently repeated at least three times. The data are reported as mean ± standard deviation (SD). The statistical significance values are defined as * *p* < 0.05, ** *p* < 0.01, *** *p* < 0.001. The statistical significance was calculated with the Mann–Whitney test performed in GraphPad Prism (San Diego, CA, USA).

## 3. Results and Discussion

This study explored the antiviral properties of natural extracts derived from *Thimelaea hirsuta*, evergreen shrubs belonging to Thymelaeaceae. Extracts from plants commonly known in the Mediterranean area, were selected for their easy accessibility and because they have been used as folk remedies for various treatment purposes. Different extracts from leaves and branches of *Thymelaea hirsuta* prepared using three increasing polarity solvents, petroleum ether, ethyl acetate (EtOAc) and acetone, (giving the extracts A, B, C, respectively) were tested in cell-based assays for cytotoxicity (CC_50_), potency (EC_50_) and spectrum of antiretroviral activity against a panel of alternative, representative positive- and negative-sense single-stranded RNA, double-stranded RNA and DNA viruses.As reported in Table 1, extracts were not able to inactivate each of these viruses, BVDV, Reo-1, Sb-1, RSV, and HSV-1, except HIV-1. While petroleum ether extracts from both leaves and branches did not inhibit HIV-1wt_IIIB_, even at the highest concentration used here (100 µg/mL). For the acetone extracts, the concentration required to reduce virus infectivity by 50% (EC_50_) was 10 µg/mL, and for the ethyl acetate extracts, the EC_50_value decreased to 0.8 µg/mL. Interestingly, as far as the cytotoxic profile is concerned, none of the six extracts werecytotoxic for exponentially growing MT-4 cells up to the highest tested concentration (CC_50_ > 100 µg/mL) as determined by the MTT assay. Concomitantly, low cytotoxicity was detected for ethyl acetate (EtOAc) and acetone extracts, with CC_50_ values mostly in the high micromolar range (100 µg/mL) in Vero-76 cells.

**Figure 1:**
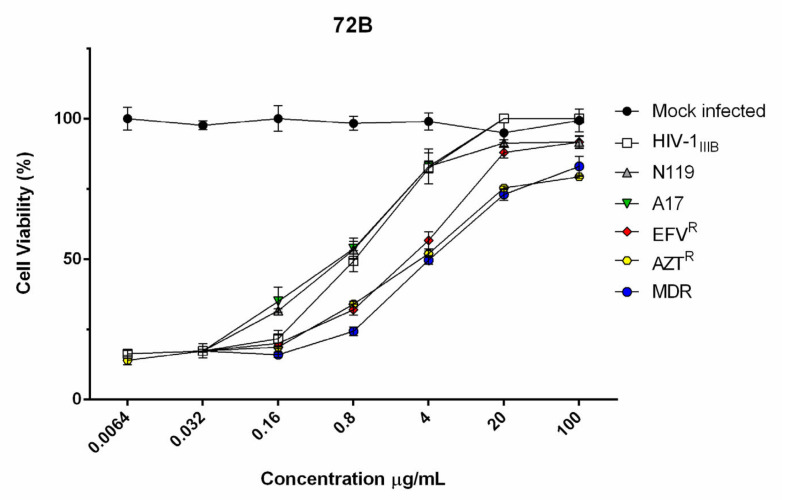
Cytotoxicity and anti HIV-1 activity of the 72B extract. To evaluate 72B antiviral activity in HIV-1 wt and drug resistant strains, several concentrations of extract (100 up to 0.0064 µg/mL) were used to inhibit wt_IIIB_ and clinically relevant RT-inhibitor resistant strains (N119, A17, EFV^R^, AZT^R^, MDR). The viability of HIV-1 infected MT-4 cells was estimated by MTT assay, 4 days post-infection. The number of live cells was expressed as a percent of mock infected, non-treated, control cells. Data are expressed as means ± SD of at least three independent measurements.

Because a critical issue in the long-term clinical management of HIV disease is the development of drug resistance that often appears during HAART therapy, reducing its effectiveness, the inhibitory activity of ethyl acetate and acetone extracts from leaves and branches were also tested against a panel of viruses possessing mutations that confer selective resistance either to nucleoside (NRTI) ornon-nucleoside (NNRTI) reverse transcriptase (RT) inhibitors. Ethyl acetate inhibitory activities were comparable with those of HIV-1 wild-type strain (Table 2 and Figure 1), making the active principles involved very promising for the future development of drugs. Then it was selected for further investigations. These results led us to further explore the inhibitory activity of the ethyl acetate extract 72B from branches.

To explore the mechanism of antiviral action, 72B was first subjected to a virus direct inactivation assay. The effect of 72B on HIV-1 inactivation infectivity, before cell infection, was investigated at 0 °C and 37 °C in an HIV-1 virucidal activity assay.

Amphotericin B, a cholesterol-depleting compound capable of inhibiting HIV-1 infectivity, was used as a reference drug because of its direct virucidal properties [41].

**Figure 2:**
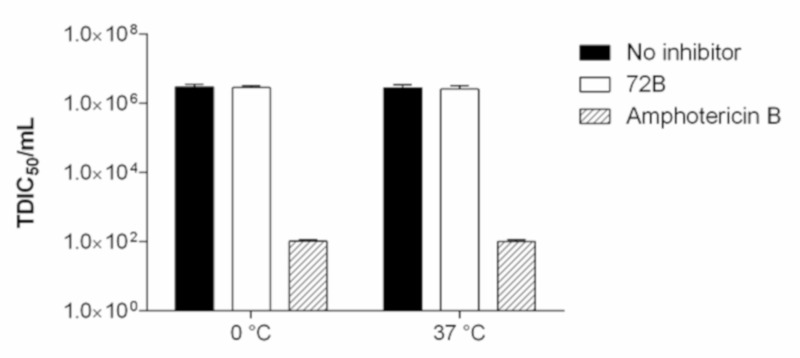
Virucidal effect (expressed as median tissue culture infectious dose (TCID_50_/mL) of 72B (30 µg/mL) against HIV-1 wt at either 0 °C or 37 °C for 2 h. Dark columns represent viral titer for viral control solution, white columns represent 72B treated solution, and line pattern columns represent amphotericin B treated solution. The results p resented were obtained from three independent experiments. Data are mean ± SD.

As shown in Figure 2, the virus titers of samples treated with 72B did not significantly differ from those determined in untreated samples, while amphotericin B reduced virus infection levels, and no significant differences between the two different temperatures were detected.

Therefore, the ethyl acetate extract did not exert its antiviral activity/inhibitory effect via the direct inactivation of HIV-1 particles.

Taken together, the results obtained in cell-based assays suggest that 72B has a mode ofaction different from drugs belonging to the major antiretroviral classes but reinforcing evidence comes from the quantitative determination of retroviral reverse transcriptase activity (RT) and protease activity (PR). Therefore, 72B was tested in an enzymatic assay aimed at evaluating their capability to inhibit HIV-1 RT reverse transcriptase activity in vitro. Interestingly, unlike the reference drug, a known RT inhibitor EFV (an HIV-1 non-nucleoside reverse transcriptase inhibitor), the extract was inactive against RT. Another critical step in the replication cycle of HIV is the proteolytic cleavage of the precursors into mature enzymes and structural proteins catalyzed by HIV PR.

**Figure 3:**
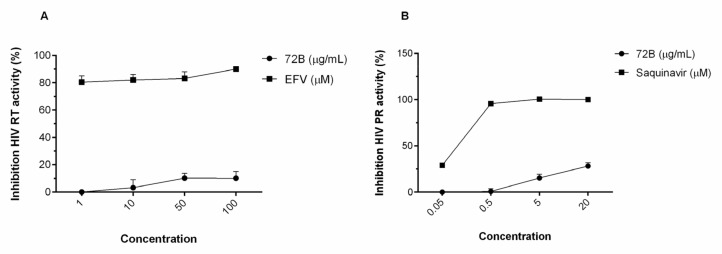
Effect of noncytotoxic concentrations of 72B on reverse transcriptase (**A**) or protease (**B**) activity. Efavirenz and saquinavir were employed as positive references in RT and PR assays, respectively. The inhibitory effect of 72B is expressed as the percentage of activity of the treated enzymes relative to control w/o treatment. The results presented were obtained from three independent experiments. Data are mean ± SD.

The effect of the 72B extract on HIV-1 protease activity was determined using Kit (Anaspec, Fremont, CA, USA). The results showed that 72B acts on a different target to PR. We demonstrated that ethyl acetate extract 72B has no RT and or PR inhibitory activity (Figure 3).

Aiming to investigate the potential anti HIV-1BaL activity, our extract was further evaluated in PM1 BaL-infected cells. HIV-1BaL is an M-tropic strain that uses CCR5 as a co-receptor. Measurement of HIV-1 p24 Gag protein in the culture supernatants collected from the infected cells indicated that it can reduce the viral titer by 80%, at a concentration of 3 µg/mL, compared to untreated infected controls. Enfuvirtide (20 µg/mL) was tested in our assay as a reference compound and showed the same trend in viral reduction [42].

Therefore, 72B proved to be active against HIV-1 with either one of the two main tropisms of T- and M-viruses that use CXCR4 or CCR5, respectively, as a co-receptor for cell entry.

Further study on the mode of action was carried out to evaluate the stage in which 72B affects the HIV replication cycle. The HIV-1 life cycle starts with viral attachment and fusion of the viral envelope with the host cell membrane.

The potential interference of extract with processes like those leading to HIV entry into susceptible cells was evaluated in co-cultures of HIV-1 chronically infected H9 cells with uninfected C8166 cells. In this assay, syncytia are generated as a result of the interaction of env-encoded glycoproteins (present on the outer membrane of chronically infected H9 cells) with the CD4 of cocultured C8166 cells. In HIV-1 infection, the appearance of syncytia is associated with a more rapid decline of CD4+ cells and progression to AIDS; therefore, agents that inhibit syncytia formation have the potential to be therapeutically useful. Many researchers have investigated strategies to inhibit both syncytium formation and HIV-1 infection [43,44,45].

**Figure 4:**
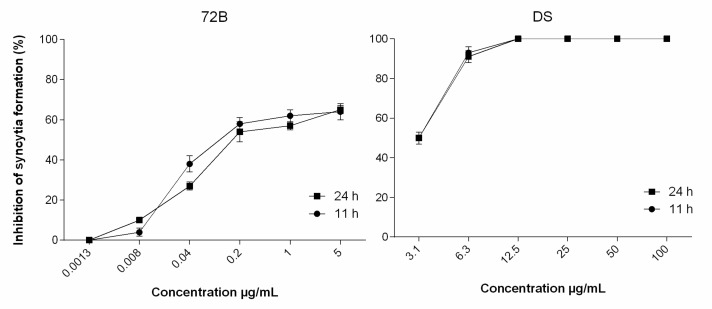
Reduction of syncytia formation. Number of syncytia at 11 (circles) and 24 (squares) hours, following treatment of C8166-H9IIIB cocultures with different concentrations of ethyl acetate branches extract (72B) and dextran sulfate (DS). The inhibition of fusion between normal C8166 cells and HIV-1_IIIB_ chronically infected H9 cells was quantified under an inverted microscope.

As shown in Figure 4, extract 72B prevented syncytia formation by more than 50% at a low concentration (1 µg/mL), similar to dextran sulfate, a sulfated polysaccharide known to prevent HIV-induced syncytium (giant cell) formation, which was used as a reference compound.

This result indicates that it interferes during early events (adsorption/attachment/fusion) in the HIV multiplication cycle triggered by the gp120-CD4 interaction, offering interesting indications for further chemical characterization of components from this extract, with the aim to design and develop more potent derivatives capable of inhibiting HIV-induced syncytia formation.

Worldwide, more than 90 percent of all young and adult HIV infections are derived from heterosexualcontact. The “feminization” of the pandemic, mainly driven by cultural, socio-economic, andbiological factors, deserves urgent attention, predominantly for the adolescent female population. Inthe absence of an effective prophylactic anti-HIV therapy or vaccine, current efforts are aimed atdeveloping intra-vaginal/rectal topical formulations of anti-HIV agents or microbicides to prevent themucosal HIV transmission.

Lactobacillus sp. are important members of the human vaginal microflora. They act as a sentinel to prevent urogenital infection and offer competitive exclusion for attachment of various pathogens, such as HIV. Lactobacilli acts like a natural “microbicide” offering the first line of defense, therefore the ethyl acetate extract must be non-toxic to their growth.

**Figure 5:**
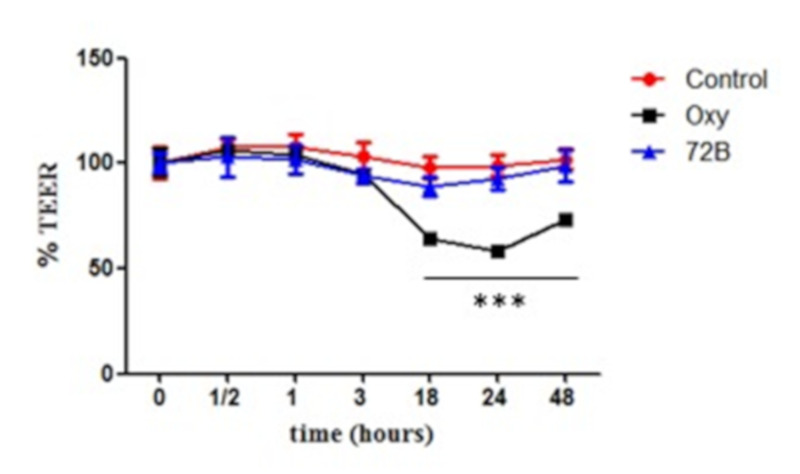
Determination of transepithelial electrical resistance (TEER). Caco-2 cell monolayers were incubated for 48 h with 72B extract (blue triangles), an oxysterol mixture as a prooxidant agent (Oxy, black squares) and positive CTRL (only solvent, red circles). The TEER value was measured at different time points to see any significant changes in monolayer permeability. Statistically significant differences are expressed (*** *p* < 0.001). Each value represents the mean ± SD of independent experiments (*n* = 3).

Incubation of various vaginal lactobacillus strains like *L. casei*, *L. paracasei*, *L. plantarum*, and *L. rhamnosus* with ethyl acetate extract up to 2000 µg/mL showed no cytotoxic effects. In the presence of our product, the viability of all Lactobacilli tested, was comparable with the untreated control. Saquinavir (protease inhibitor) and TMC 120 (promising microbicide) were used as HIV-1 reference inhibitors. In the female genital tract, low pH, the mucosal immune system, an intact epithelial surface and the presenceof lactobacilli represents a natural hurdle for HIV. An efficacious and low-cost method to assess the safeness of a potential microbicide could be to test its cytotoxicity against lactobacilli. HIV is mainly transmitted by sexual contact and a critical issue for the prevention of sexual transmission is the integrity of the reproductive tract epithelium. The effect of the active extract on epithelial cell monolayer integrity was measured using a Caco-2 model, each day, as transepithelial electrical resistance (Figure 5), by a voltmeter, until resistance reached a plateau. Then, a non-toxic concentration of 72B (30 µg/mL) was added and resistance (TEER) was monitoredat intervals until 48h. Only solvent (Control) and an oxysterols mixture (Oxy, 60 µM), which induce prooxidant and proinflammatory effects on membrane lipids [46], were used as positive and negative controls, respectively.

It was observed that the concentration of ethyl acetate extract 72B did not affect the TEER over the time of the experiment, keeping values similar to those of the untreated cells. Therefore, it may be suggested as a suitable candidate for topical application and these findings prompt us to conduct further studies on new in vitro barrier model systems.

## 4. Conclusions

Historically, natural products, either as pure compounds or as standardized plant extracts, have been asource of inspiration for the development of new drugs. In recent decades, they are a promisingsource of new therapeutic agents. In our studies, ethyl acetate extracts from *Thymelaea hirsuta* turned out to have interesting activity against HIV-1. Extract 72B was not virucidal, but it was potently active against resistant strains. However, it was not able to inhibit the RT and protease enzymatic functions. The considerable inhibitory effect on the syncytia formation in co-cultures showed that 72B inhibits an early event in the replication cycle ofHIV. The preclinical safety profile of this extract showed no adverse effect on the growth ofLactobacilli, and non-toxic concentration of the extract did not influence the TEER in intestinal epithelial cells. These findings are encouraging and further safety and efficacy in vitro and in vivo studies will be performed to identify the chemical constituents of these extracts and to better define the mechanism of action.

## Figures and Tables

**Table 1:** Cytotoxicity and antiviral activity of *Thymelaea hirsuta* extracts against representatives of ssRNA^+^ (HIV-1, BVDV, Sb-1), ssRNA^-^ (RSV, VSV), dsRNA (Reo-1) and dsDNA (HSV-1) viruses. °

Compounds	MT-4	HIV-1_IIIB_	MDBK	BVDV	BHK	Reo-1	Vero-76	Sb-1	RSV	VSV	HSV-1
	CC_50_^a^	EC_50_^b^	CC_50_^c^	EC_50_^d^	CC_50_^e^	EC_50_^f^	CC_50_^g^	EC_50_^h^	EC_50_^j^	EC_50_^k^	EC_50_^l^
71 (Leaves)											
A	>100	>100	>100	>100	>100	>100	>100	>100	>100	>100	>100
B	>100	0.8 ± 0.1	>100	>100	>100	>100	84	>84	>84	>84	>84
C	>100	5	>100	>100	>100	>100	92	>92	>92	>92	>92
72 (Branches)											
A	>100	>100	>100	>100	>100	>100	>100	>100	>100	>100	>100
B	>100	0.8 ± 0.1	>100	>100	>100	>100	84	>84	>84	>84	>84
C	>100	5	>100	>100	>100	>100	94	>94	>94	>94	>94

A (*petroleum ether*); B (*ethyl acetate*); C (*acetone*). Data represent mean values ± SD for three independent determinations. For values where SD is not shown, variation among duplicate samples was less than 15%. ^a^Compound concentration (µg/mL) required to reduce the proliferation of mock-infected MT-4 cells by 50%, as determined by the MTT method. ^b^Compound concentration (µg/mL) required to achieve 50% protection of MT-4 cells from HIV-1 induced cytopathogenicity, as determined by the MTT method. ^c^Compound concentration (µg/mL) required to reduce the viability of mock-infected MDBK cells by 50%, as determined by the MTT method. ^d^Compound concentration (µg/mL) required to achieve 50% protection of MDBK cells from BVDV-induced cytopathogenicity, as determined by the MTT method. ^e^Compound concentration (µg/mL) required to reduce the viability of mock-infected BHK cells by 50%, as determined by the MTT method. ^f^Compound concentration (µg/mL) required to achieve 50% protection of BHK cells from Reo-1-induced cytopathogenicity, as determined by the MTT method. ^g^Compound concentration (µg/mL) required to reduce the viability of mock-infected Vero-76 cells by 50% as determined by the MTT method. ^h^Compound concentration (µg/mL) required to reduce the plaque number of Sb-1 by 50% in Vero-76 monolayers. ^j^Compound concentration (µg/mL) required to reduce the plaque number of RSV by 50% in Vero-76 monolayers. ^k^Compound concentration (µg/mL) required to reduce the plaque number of VSV by 50% in Vero-76 monolayers. ^l^Compound concentration (µg/mL) required to reduce the plaque number of HSV-1 by 50% in Vero-76 monolayers.

**Table 2:** Cytotoxicity and antiviral activity of *Thymelaea hirsuta* extracts, and reference compounds, against HIV-1, and its NNRTI- (N119, A17, EFV^R^) and NRTI- (AZT^R^, MDR) resistant mutants.

Compounds	MT-4	HIV-1_IIIB_	N119(Y181C)	A17 (K103N, Y181C)	EFV^R^ (K103R, V179D, P225H)	AZT^R^ (67N, 70R, 215F, 219Q)	MDR (74V, 41L, 106A, 215Y)
	CC_50_^a^	EC_50_^b^					
71 (Leaves)							
A	>100	>100	-	-	-	-	-
B	>100	0.8 ± 0.1	0.35 ± 0.005	0.4 ± 0.05	0.7 ± 0.1	3 ± 0.01	4 ± 0.01
C	86	10	9	8	10	>86	15
72 (Branches)							
A	>100	>100	-	-	-	-	-
B	>100	0.8 ± 0.1	0.5 ± 0.003	0.4 ± 0.01	2 ± 0.05	3 ± 0.03	4 ± 0.03
C	70	10	9	9	13	>70	16
References *							
Azidothymidine	45	0.02 ± 0.003	0.02 ± 0.003	0.01/ ± 0.002	0.02 ± 0.003	7.0 ± 0.05	0.65 ± 0.01
Efavirenz	40	0.003 ± 0.0003	0.02 ± 0.003	0.10 ± 0.009	13.0 ± 2.1	0.0035 ± 0.0003	0.01 ± 0.05
Nevirapine	>100	0.08 ± 0.01	8.0 ± 08	70	100	0.4 ± 0.1	3.0 ± 0.6

A (*petroleum ether*); B (*ethyl acetate*); C (*acetone*); Data represent mean values ± SD for three independent determinations. For values where SD is not shown, variation among duplicate samples was less than 15%. ^a^Compound concentration (µg/mL) required to reduce the viability of mock-infected MT-4 cells by 50%, as determined by the MTT method. ^b^Compound concentration (µg/mL) required to achieve 50% protection of MT-4 cells from HIV-1 induced cytopathogenicity, as determined by the MTT method. * Reference compounds: CC_50_ and EC_50_ are in µM.
